# Seizure in Morphea: A Case Report of Parry-Romberg Syndrome

**DOI:** 10.7759/cureus.65210

**Published:** 2024-07-23

**Authors:** Veerasivabalan S, Hema Murugesan, Kalpana Ramanathan, Chandrasekar Selvaraj

**Affiliations:** 1 Department of General Medicine, Stanley Medical College and Hospital, Chennai, IND; 2 Department of Rheumatology, Stanley Medical College and Hospital, Chennai, IND

**Keywords:** parry-romberg syndrome, morphea, progressive hemifacial atrophy, epilepsy, seizure, en coup de sabre

## Abstract

Parry-Romberg syndrome is a rare neurocutaneous disease characterized by progressive hemifacial atrophy. We present the case of a 14-year-old, a known case of linear morphea, who presented with seizure and on evaluation was diagnosed with Parry-Romberg syndrome. It causes a profound impact on aesthetic well-being and has a significant psychosocial morbidity. This case report aims to highlight the effective multidisciplinary team approach involving a rheumatologist, dermatologist, neurologist, and ophthalmologist which ultimately culminated in the meticulous management of the disease in our patient.

## Introduction

Parry-Romberg syndrome is a rare neurocutaneous disease characterized by progressive, disfiguring atrophy of the skin and the underlying subcutaneous tissue, muscle, and bone on one side of the face [1-3]. The prevalence is estimated to be at 1 in 700,000 individuals. It may go on to involve the eyes in the form of enophthalmos, madarosis, ocular telangiectasias, uveitis, ocular myopathy, and heterochromia [1]. The dermatological manifestations include the characteristic linear scleroderma or “en coup de sabre,” resembling a strike with a sword [2,4]. The dental abnormalities include hemimasticatory spasm, hemiatrophy of the tongue, and tooth malocclusion [3]. The central nervous system manifestations include migraine, hemifacial spasms and pain, trigeminal neuralgia, visual field defects, and seizures [5]. Diagnosis of Parry-Romberg syndrome is predominantly made on clinical grounds aided by dermatopathological and radiological investigations such as an MRI of the brain when the patient presents with a seizure, as in our case report [1,4].

## Case presentation

**Figure 1:**
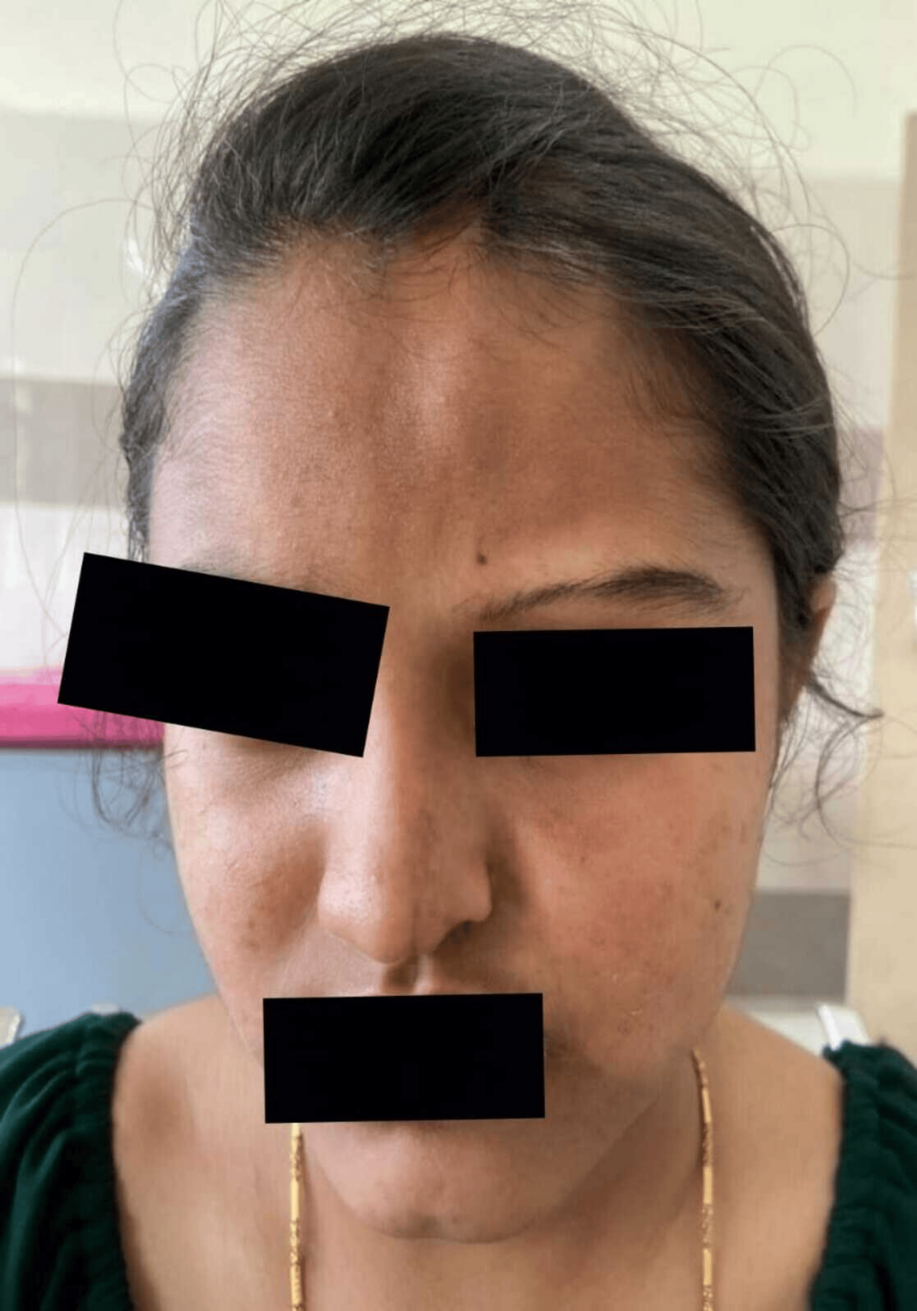
An irregular, disfiguring, and atrophic plaque extending from the left lateral side of the nose and along the left outer canthus of the eye to involve the hairline in the frontal region of the scalp. Thinned-out left eyebrows can be noted.

**Figure 2:**
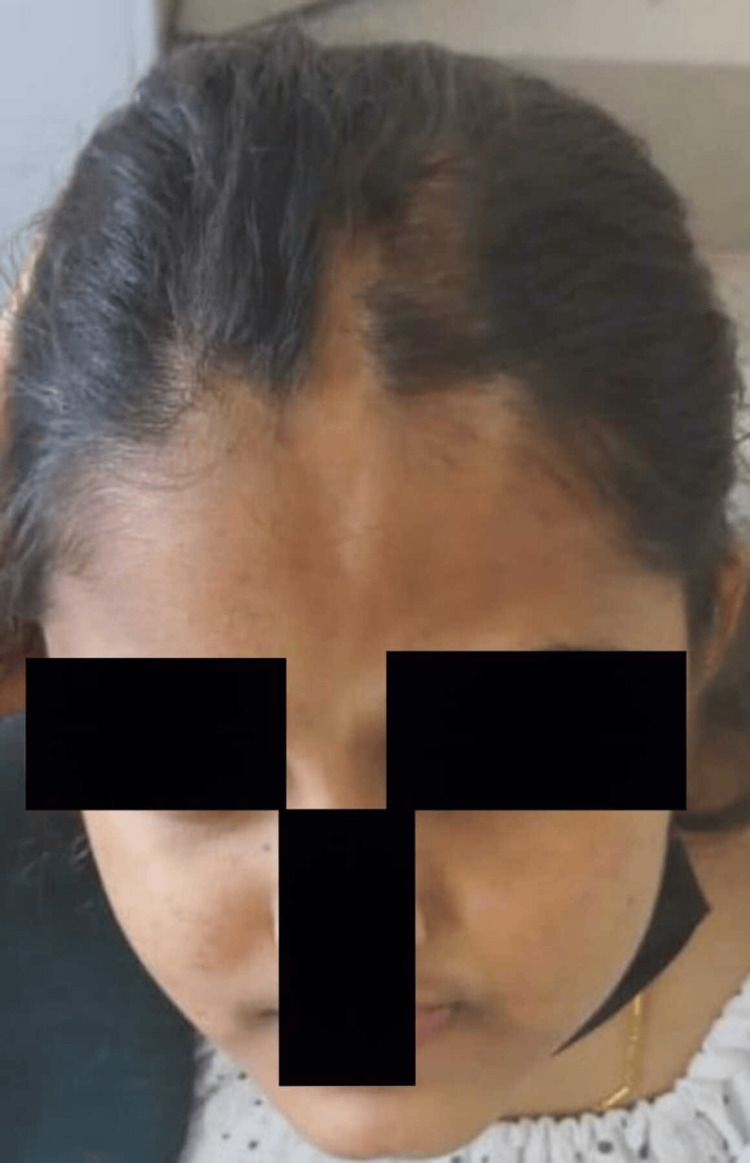
Scarring alopecia over the frontal region of the scalp.

**Figure 3:**
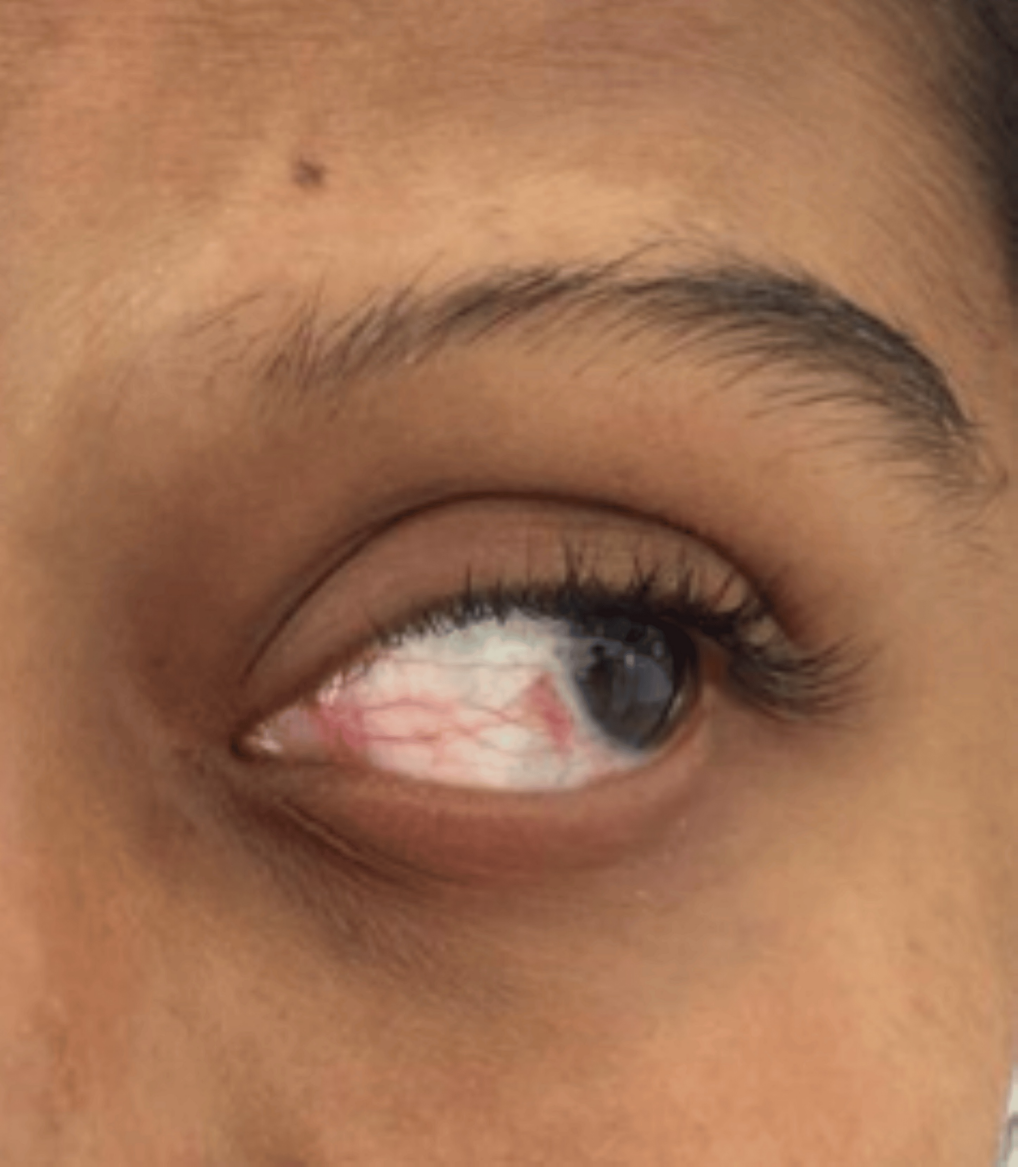
Left eye telangiectasia.

A developmentally normal 14-year-old girl, born out of a non-consanguineous marriage, with good academic performance, presented to our hospital with complaints of headache, blurring of vision, and an episode of seizure. There was no history of sensory or autonomic system involvement. There was no prior history of fever, ear discharge, exposure to toxins, insect bite, envenomation, altered behavior, trauma, and bleeding manifestations. There was no history of neonatal seizure. She had regular menstrual cycles. On examination, the vital signs were normal. Capillary blood glucose was 123 mg/dL. Central nervous system examination revealed normal higher mental function with normal motor, sensory, and autonomic functions. Her speech and memory were intact. Other system examinations were normal. Examination of the face showed atrophic plaque on the left side of the face (Figure 1), scarring alopecia over the frontal region of the scalp (Figure 2), and left eye telangiectasia (Figure 3).

An irregular and disfiguring atrophic plaque was noted over the left side of the face. It was extending from the left lateral side of the nose vertically up to the hairline in the frontal region of the scalp. The left eyebrows and eyelashes were thinned out. Her history revealed that she had been diagnosed with linear scleroderma at the Department of Dermatology in 2020. The patient was treated with topical steroids and topical immunosuppressive therapy. Despite topical therapy, the skin lesion progressed to involve the underlying muscle and the frontal bone for which she was switched over to systemic immunosuppressants (tablet methotrexate). Further, to enhance the aesthetic well-being of the patient, serial doses of intralesional triamcinolone injection, hyaluronic acid fillers, and autologous platelet-rich plasma were injected over the linear atrophic plaques over the frontal area of the scalp and the tip of the nose.

**Figure 4:**
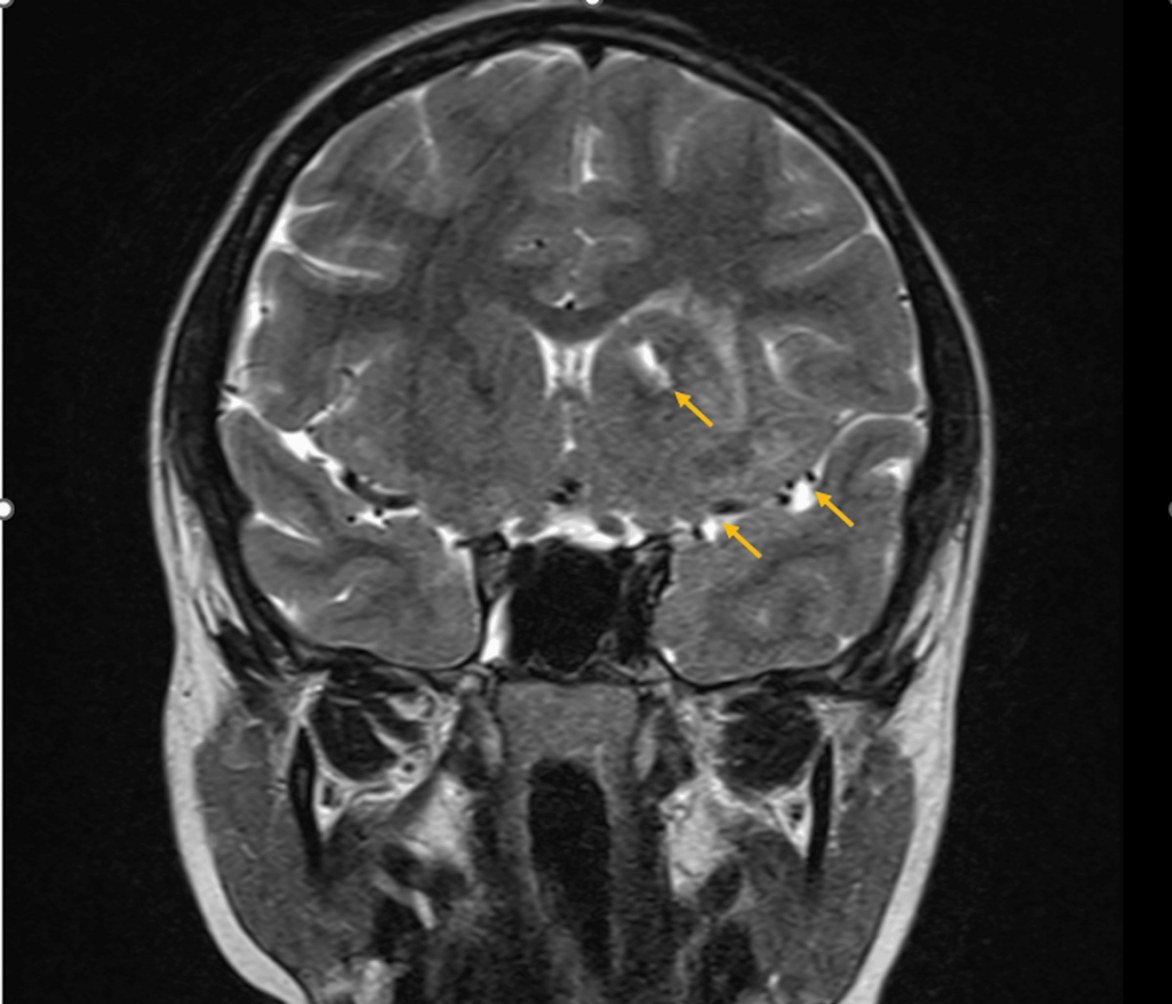
MRI of the brain showing ill-defined T2-weighted hyperintensities with tiny cystic spaces, multiple calcifications, and microhemorrhages involving the left gangliocapsular region, left parietooccipital, and left temporal cortex (yellow arrows).

**Figure 5:**
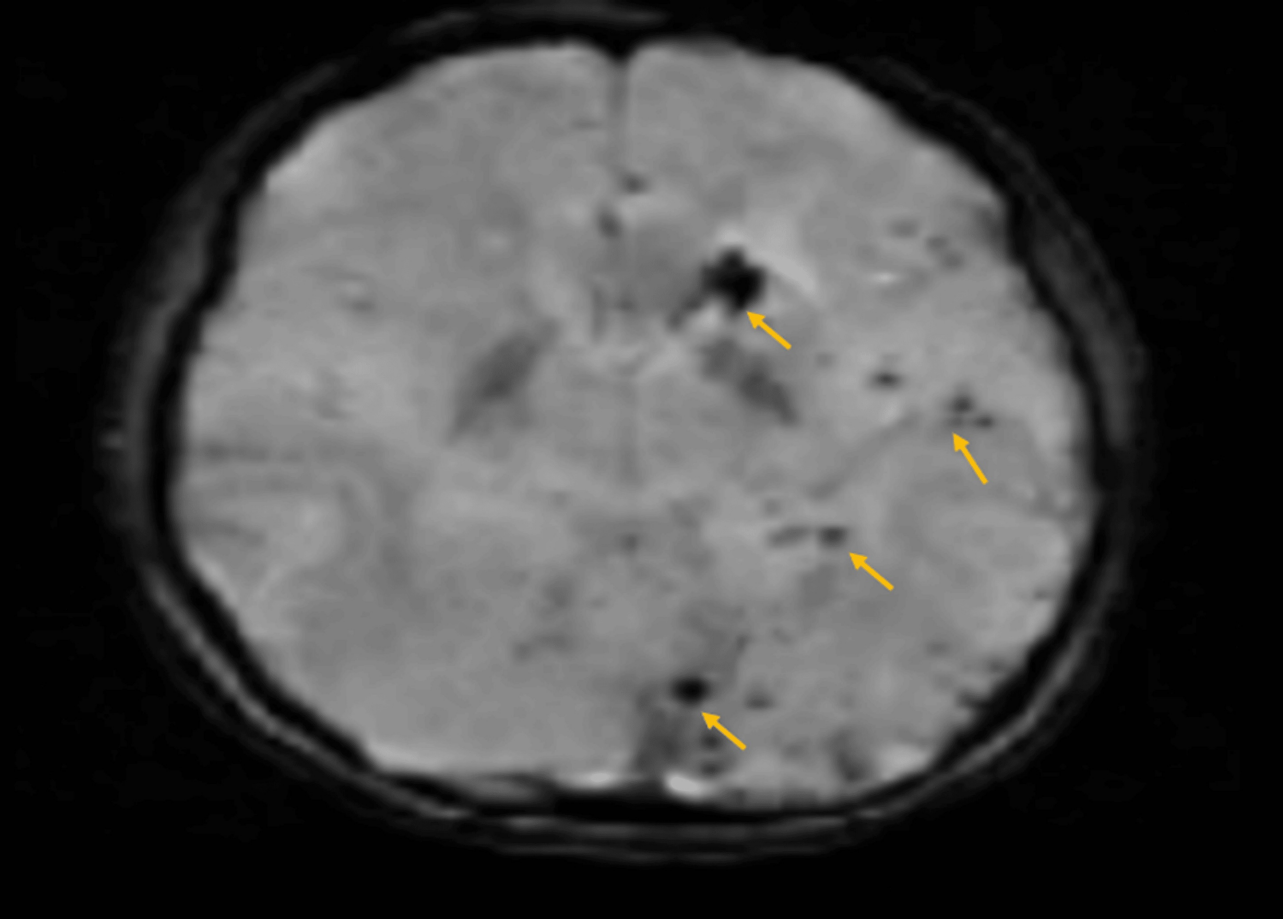
MRI (susceptibility-weighted imaging) of the brain showing tiny cystic spaces, multiple calcifications, and microhemorrhages involving the left gangliocapsular region, left parietooccipital, and left temporal cortex (yellow arrows).

During the current admission to the hospital, the patient presented with a right focal seizure. MRI of the brain showed ill-defined T2-weighted hyperintensities with tiny cystic spaces, multiple calcifications, and microhemorrhages involving the left gangliocapsular region, left parietooccipital, and left temporal cortex (Figures 4, 5). Three-dimensional time-of-flight MR angiography for the circle of Willis revealed no evidence of stenosis, aneurysm, or vascular malformation. Video electroencephalography (EEG) showed a focal slowing in the left temporo-occipital region, normal awake study, and well-formed sleep patterns with no recorded clinical event during the study. The ophthalmological assessment showed a normal fundus. Humphrey’s visual field testing revealed the right homonymous hemianopia correlating with the MRI finding of the involvement of the left parietooccipital cortex of the brain. A comprehensive laboratory investigation showed a normal complete blood count, normal renal function test, and normal liver function test. The erythrocyte sedimentation rate was 5 mm/hour, the C-reactive protein was negative, and the antinuclear antibody profile was negative. Hence, the diagnosis of Parry-Romberg syndrome was made based on the MRI and EEG findings in the background of linear morphea. Opinions from a neurologist and a rheumatologist were obtained and the patient was started on antiepileptics (tablet levetiracetam and carbamazepine) and tablet mycophenolate mofetil for immunosuppression. The patient is doing well with no further seizure episodes.

## Discussion

Parry-Romberg syndrome is a neurocutaneous disease characterized by progressive hemifacial atrophy. It is a multisystem disorder, and the etiology of the disease remains multifactorial, including genetic ambiguity, vasculopathy, autoimmunity, and microbial causation which are set to operate under an inflammatory background [1-4]. The prevalence is estimated to be at 1 in 700,000 individuals. Although Parry-Romberg syndrome becomes apparent usually in the first decade of life or early during the second decade, it can also manifest in adulthood. There is a bimodal peak of occurrence of the illness: one between 2 and 14 years of life and a second peak in the fifth decade of life. The disease demonstrates a female preponderance with an overall female-to-male ratio of 4:1 [4].

This syndrome encompasses protean clinical manifestations. The extracutaneous involvement has a greater overall disease burden and follows the skin disease in general.

The oral manifestations include dental malocclusion due to abnormal bone growth, hemimasticatory spasm, and hemiatrophy of the tongue [3]. The varied ocular manifestations are enophthalmos, madarosis, ocular telangiectasias, heterochromia, uveitis, ocular myopathy, and strabismus [4]. The neurological manifestations include headache, migraine, hemifacial spasm, trigeminal neuralgia, seizures, visual field defects, movement disorders, peripheral neuropathy, and neuropsychiatric symptoms. Both seizures and headaches can be the presenting features of Parry-Romberg syndrome [5-8].

The skin sign is named “en coup de sabre,” resembling a strike with a sword. It is important to understand that both Parry-Romberg syndrome and “en coup de sabre” belong to the same spectrum of disease called localized scleroderma.

The rare manifestations in our case, as discussed above, include right focal seizure at presentation, madarosis, ocular telangiectasia, and right homonymous hemianopia.

Most patients tend to have normal neurodevelopment until seizure manifests in the first decade of life. The incidence of seizure is likely to be estimated at 11% [5]. The most commonly encountered seizure variant is focal seizure with impaired awareness, as presented in our case. The other varieties include generalized tonic-clonic seizures and epilepsia partialis continua. The seizure tends to become refractory in 40% of the cases [5]. Antiepileptic drugs and immunosuppressive drugs form the cornerstone of management of the seizure [5-8]. In patients with refractory epilepsy, surgical hemispherectomy may be considered [5,7]. The neurological sequelae in the form of residual hemiparesis and cognitive impairment add to the burden of the disease.

The differential diagnosis includes Rasmussen encephalitis, Dyke-Davidoff-Masson syndrome, Goldenhar syndrome, and Barraquer-Simons syndrome [2,6,7].

It is important to highlight at this juncture that localized morphea does not transit to systemic sclerosis in general. Furthermore, Parry-Romberg syndrome has a waning and waxing clinical course with frequent relapses and remission, thereby affecting the aesthetic well-being of the patient, fueling the functional disability, and carrying psychosocial morbidity.

Diagnosis of Parry-Romberg syndrome is made on clinical grounds and aided by dermatopathological and radiological investigations such as MRI of the brain. A dermatopathological examination of the localized scleroderma-morphea would reveal dermal sclerosis, collagen homogenization, dermal thickening, inflammatory cell infiltration, and loss of skin appendages [4]. MRI of the brain may show hemiatrophy, calcifications, microhemorrhages, cortical dysplasia, T2 hyperintense signal mainly in the subcortical white matter, leptomeningeal enhancement, vascular malformations, and vascular aneurysm [1,6-8].

The treatment is directed toward the specific symptoms that are apparent in each affected individual. It is generally a multidisciplinary team approach that comes into play for the meticulous management of the rare disease. Cosmetic procedures such as fat, bone, cartilage grafts, and silicone implants can be considered [3,7]. Topical tacrolimus 0.1% ointment, topical vitamin D derivatives such as calcipotriene 0.005% and calcipotriol 0.005% ointments, and topical imiquimod 5% ointments in conjunction with phototherapy can be used for active plaque-type morphea [4]. Systemic therapy either with methotrexate alone or in combination with systemic steroids is recommended for deep, generalized, pan-sclerotic, or progressive linear morphea and Parry-Romberg syndrome. Mycophenolate mofetil can be used in patients who show relapse, refractoriness, and intolerance to methotrexate therapy [4]. In our case, the patient was treated with serial doses of injection triamcinolone, hyaluronic acid, and autologous platelet-rich plasma which improved the aesthetic well-being of the patient and helped her cope up with the functional and psychosocial constraints of her illness. The seizure component of this disease can be well managed with antiepileptics. Immunosuppression is the goal and can be achieved with a weekly regimen of tablet methotrexate given as a single oral dose of 15-25 mg/week and tablet mycophenolate mofetil at a dose of 2 g/day for methotrexate refractory disease [4].

## Conclusions

Parry-Romberg syndrome is a disfiguring disease of the face causing psychosocial morbidity. Patients with Parry-Romberg syndrome can lead a near-normal life when a prompt diagnosis is made with a thorough neurological examination, MRI, and EEG in patients presenting with hemifacial atrophy. It is important to consider the sporadic nature and the rarity of Parry-Romberg syndrome. There is neither a standardized treatment protocol nor a cure for this illness. The treatment is directed toward the specific symptoms that are apparent in each affected individual. It is the multidisciplinary team approach that comes into play during the active progression of the disease. It is prudent to initiate immunosuppressive and anticonvulsant therapy at diagnosis to decrease the morbidity of the illness.
